# Strategies for Greener Hospital Operating Rooms

**DOI:** 10.1289/ehp.120-a306a

**Published:** 2012-08-01

**Authors:** Carol Potera

**Affiliations:** Carol Potera, based in Montana, has written for *EHP* since 1996. She also writes for *Microbe*, *Genetic Engineering News*, and the *American Journal of Nursing*.

Hospitals produce prodigious amounts of waste each day, with operating rooms (ORs) and labor and delivery units contributing the majority.^1^ Yoan Kagoma and colleagues at the University of Western Ontario measured the waste from total knee replacements in Canadian hospitals and found this procedure alone produced about 450 tons of waste over the period 2008–2009.^2^ Astounded by these results, they analyzed 65 published studies about the environmental impact of OR waste and practices, and concluded in the 4 June 2012 issue of the *Canadian Medical Association Journal* that the “provision of health care is not benign, and many of the interventions used to save lives [adversely] affect the environment.”^3^ However, they also uncovered several green practices that are easily implemented and can save money without compromising patient care.

Some green strategies are nontechnical; one example is carefully sorting OR waste. Managing biohazard waste—which includes body fluids, tissues, and anything they touch, such as sponges, gowns, and gloves—requires autoclaving, incineration, and other energy-intensive processes. Yet up to 90% of waste tossed into biohazard containers is nonhazardous,^4^ partly because staff are overly cautious and partly because it’s easier to put all waste in one container instead of taking time to sort it, Kagoma says. In 2010 more attention to segregation at one hospital in Pittsburgh, Pennsylvania, reduced by half the amount of biohazard waste processed and saved $89,000 in associated costs.^5^ Reducing biohazard waste also means less is incinerated, a process that produces toxic nitrous oxide, polychlorinated biphenyls, furans, and dioxins.^6^

A more technical solution sequesters OR anesthetic gases such as isoflurane. This and other halogenated ethers are up to 3,760 times more powerful at trapping heat than carbon dioxide, and up to 95% of anesthetics are vented to the outside.^7^ By one estimate, the yearly anesthetic gas emissions from a midsize hospital are equivalent to carbon dioxide emissions from 1,200 automobiles.^7^

ORs across Ontario now capture 100% of anesthetic gases with Deltasorb® canisters, developed by Blue-Zone Technologies in Concord, Ontario. An inner crystalline matrix traps only halogenated anesthetics, which the company recovers and recycles.^8^ Chemical engineer Dusanka Filipovic, co-inventor of the technology with anesthesiologists at the University of Toronto, says that in addition to keeping greenhouse gases out of the atmosphere, recycling ensures a secure supply of anesthetics, many of which are in short supply.^9^ Just a few companies manufacture the anesthetics used by ORs in developed countries. “We improve supply security by recycling them,” Filipovic says.

A quarter of hospitals reprocess single-use OR devices such as ultrasonic scalpels and trocars (used to puncture the skin to drain fluids).^10^ In the United States, reprocessed devices are decontaminated and tested for proper functioning before reuse by commercial reprocessors under Food and Drug Administration guidelines. One reprocessing company saved its customers more than $138 million and diverted 2,150 tons of waste from landfills in 2008.

Although reprocessing requires energy and cleaning agents, “these items are very functional and should be recycled instead of mining new metals or making new plastics to replace them,” says Kagoma. Stryker Sustainability Solutions, a major reprocessing company in Tempe, Arizona, uses all biodegradable cleaning agents and recycles OR devices that cannot be reprocessed. “Reprocessed items cost about half as much as new counterparts, because we’re not creating new raw materials,” says Emily Hansen, associate marketing director at Stryker.

**Figure:**
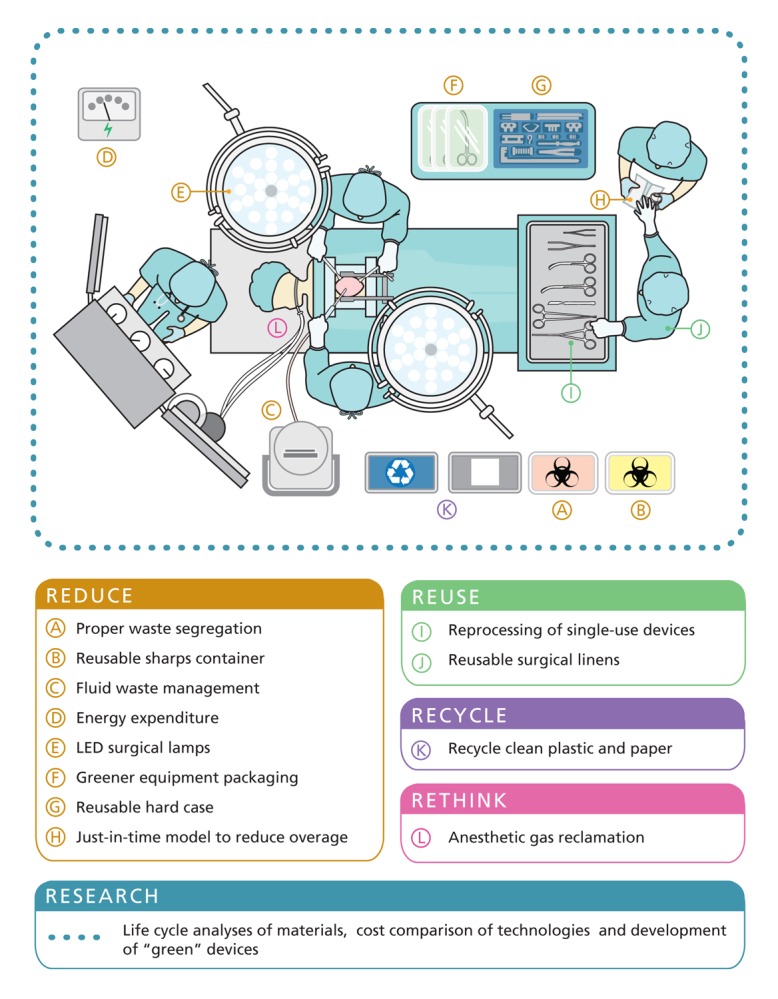
© Janice Yau, doi:10.1503/cmaj.112139

“Overage” also plagues ORs, which often use prepackaged supply kits for individual surgeries. The kit may include up to 100 items that are set out for surgery but remain unused, and once the outer packaging of the kit is open, the unused items must be discarded. By redesigning surgical kits to include only necessary equipment, a Minneapolis, Minnesota, medical center avoided more than 2.5 tons of waste and saved $81,000 in 2010.^5^

Kagoma’s team tackled overage another way: by founding Operation Green, which collects unused sponges, gloves, gauze, syringes, and other items from ORs in Ontario.^11^ The items are donated to the nonprofit International HOPE Canada and sent to hospitals and clinics in developing countries. Operation Green is modeled after REMEDY (Recovered Medical Equipment for the Developed World), a 20-year-old project at Yale University that has donated more than 50 tons of recovered medical materials since it began.^12^ “It only takes a few individuals who are interested in making a change,” says Kagoma.
